# The promotion of homebased physical activity for people with lung cancer and cachexia, a qualitative study of healthcare professionals, patients and carers

**DOI:** 10.1007/s11764-023-01376-3

**Published:** 2023-04-24

**Authors:** Nichola Gale, Jane Hopkinson, David Wasley, Anthony Byrne

**Affiliations:** 1 School of Healthcare Sciences, College of Biomedical and Life Sciences, Cardiff University, Heath Park, Cardiff, CF14 4XN UK; 2 School of Healthcare Sciences, Cardiff University, 13.10, 13th Floor, Eastgate House, 35 - 43 Newport Road, CF24 0AB Cardiff, UK; 3 Cardiff School of Sport, Cardiff Metropolitan University, Cardiff, CF23 6XD UK; 4 Cardiff and Vale University Health Board, University Hospital Llandough, Penlan Road, Cardiff, CF64 2XX UK

## Abstract

**Purpose:**

There is some evidence of the benefits of physical activity (PA) in patients with lung cancer; however, there is a lack of understanding of acceptable PA for patients with established cachexia and how to facilitate sustainable behaviour change to promote PA. Therefore, this study explored the views of healthcare professionals (HP), patients with lung cancer and cachexia, and their carers on preferences for, barriers and facilitators of homebased PA.

**Methods:**

This qualitative study involved ten telephone interviews with HPs and face-to-face interviews with seven patients with lung cancer and cachexia and their carers. Interviews were transcribed and analysed thematically. The Capability, Opportunity, Motivation and Behaviour (COM-B) model was used as a framework for the thematic cross-group analysis.

**Results:**

The types of homebased PA suggested by patients with lung cancer and cachexia (*n* = 7), their carers (*n* = 7) and HPs (*n* = 10) were functional, flexible, individualised and initially of short duration and low intensity. PA was influenced by themes within physical and psychological Capability, physical and social Opportunities as well as automatic and reflective Motivation.

**Conclusion:**

Based on a behaviour change theory, principles to promote homebased PA were developed. These principles need to be integrated into tools to promote PA in people with lung cancer and weight loss.

**Implications for Cancer Survivors:**

The application of the proposed principles by clinicians will promote physical activity, enhancing the function and wellbeing of patients with lung cancer and reducing burden on carers.

**Supplementary Information:**

The online version contains supplementary material available at 10.1007/s11764-023-01376-3.

## Introduction

Cancer is an increasing cause of morbidity and mortality, resulting in a significant burden on patients, carers, health and social care. Lung cancer is one of the most common types with a particularly poor prognosis and many patients present with advanced disease [1]. Approximately 50% of patients with advanced cancer have loss of weight and muscle, with cancer cachexia defined as greater than 5% weight loss, or 2% in individuals with a low body-mass index [BMI] < 20 kg/m^2^, in the last 6 months [2]. Such patients have poor outcomes including impairment of daily activities, independence and quality of life (QoL) [3].

Physical activity (PA) is any muscle movement that requires energy, and includes exercise, which is defined as planned, structured, repetitive activity intended to improve or maintain physical fitness [4]. There is evidence that exercise can improve QoL in patients with cancer [5]. However, there are limited studies in lung cancer, and in patients with advanced cancer, results have not been consistent. Some PA interventions have improved function and symptoms [6] while others have had limited benefits [7]. The majority of studies have included programmed exercise, in a group or hospital setting, but reported high dropout rates as many individuals were unable to maintain the required exercise frequency and/or intensity [8]. A pilot randomised controlled trial (RCT) of a homebased walking programme (*n* = 40) using a mobile application demonstrated increases in PA, physical role functioning and a trend of improved dyspnoea in patients with stage 3 and 4 lung cancer [9]. However the majority of the patients in these studies were also relatively high functioning, with under-representation of patients with established weight loss. A questionnaire study exploring exercise preferences, motivations and self-efficacy showed that patients with cancer cachexia (lung or gastrointestinal) saw the value of PA, with preferences for low-intensity structured activities, undertaken individually, at home [10].

**Fig. 1:**
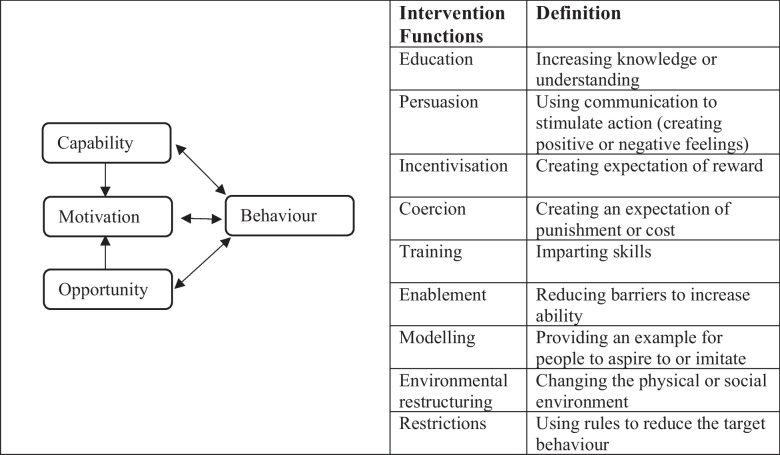
The COM-B system & Behaviour Change Wheel with definition of intervention functions adapted from Mitchie et al. 2011 [16]

There is a lack of guidance on the types of PA which are acceptable to patients, and how to promote PA to people with lung cancer and in particular those with cancer cachexia [11]. Behaviour Change Techniques (BCTs) have been associated with successful PA improvements in cancer but no studies in lung cancer were included, so specific BCTs have yet to be identified [12]. The Behaviour Change Wheel (BCW; Fig. 1) is a multiphase process guide for developing complex behaviour change interventions. It comprises the COM-B model which suggests that behaviour (B) is influenced by Capability (C) (including psychological/ physical), Opportunities (O) (including social and physical), and Motivation (M) (including automatic and reflective). These can be mapped to intervention functions: approaches that can be used to change behaviour [13]. The intervention functions can be linked to BCTs which are specific actions that will bring about change [14]. Involving users and providers in developing interventions is a sustainable method to change or develop services [15]. Therefore, the purpose of this study was to explore the views of health professionals, patients with lung cancer and cachexia and their carers on suitable homebased PA and explore barriers and facilitators, to inform the promotion of homebased PA. The COM-B Behavioural Change Wheel was used to provide a framework for analysis to categorise and develop principles to promote PA.

## Method

### Design

**Fig. 2:**
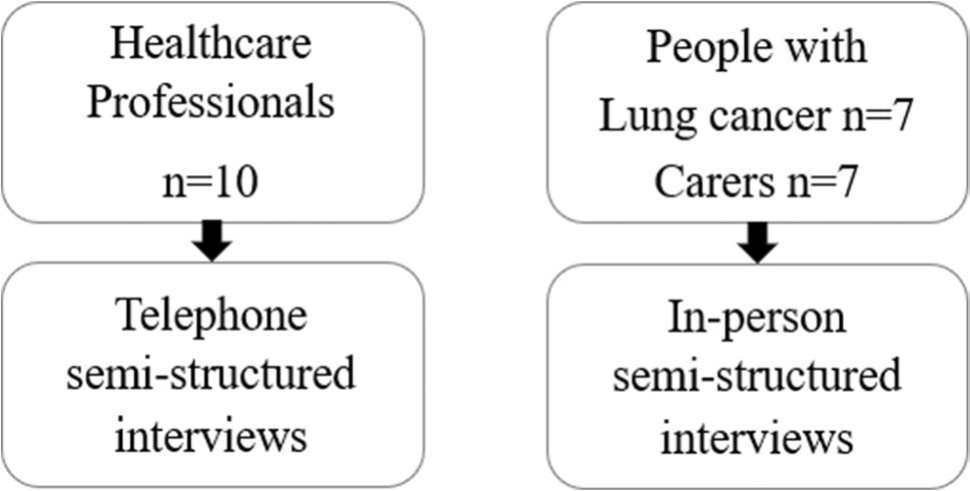
Participant diagram

This qualitative study included semi-structured telephone interviews with healthcare professionals and face-to-face interviews with patients with lung cancer and cachexia and their carers, where possible. A schematic of the study is presented in Fig. 2.

### Participants

Healthcare professionals (*n* = 10) with at least 2 years of experience in cancer care were purposively recruited in person from the Council for Allied Health Professional Research events and existing networks via email. The sample size was based on a systematic review suggesting 9–17 participants are required for data saturation [17]. Individuals were invited to participate in telephone interviews with written informed consent obtained before the interview. The interviews were conducted by (NG (PhD) Physiotherapy Lecturer with previous experience undertaking mixed methods research) using a topic guide developed by the research team and informed by behaviour change theory and containing open-ended questions with the objective to identify suitable homebased physical activities, risks and benefits and strategies for promoting physical activity (Appendix 1). Ethical approval was gained from the University Ethics Committee to interview HPs.

Patients (*n* = 7) and their carers (*n* = 7) were purposively recruited from NHS Palliative clinics in the UK. The planned sample size was up to 10 based on the literature [17] and considering the burden on participants meeting inclusion/exclusion criteria. Potential participants were identified by members of the usual care team and were not known to the researcher. Inclusion criteria were adults with a confirmed diagnosis of lung cancer and life expectancy of > 8 weeks, with cachexia (defined as weight loss of at least 5%, or a BMI of < 20 and any weight loss in the preceding 6 months). Exclusion criteria were patients with musculoskeletal or neurological disorders which would compromise mobility. Interviews were arranged to take place at a time and place most convenient to the participants the majority were in the participants own home, with two undertaken at the University. The interviews were conducted by NG using a topic guide containing open-ended questions developed by the research team and informed by behaviour change theory with the objective to identify suitable homebased activities and explore barriers and facilitators for homebased PA. The purpose of the research was explained to participants and informed consent gained prior to the interview. National Research Ethics Committee (NREC IRAS245779) approval was gained for patient and carer interviews.

### Data handling and analysis

**Fig. 3:**
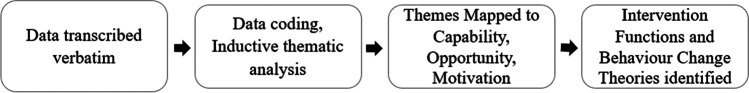
Analytical process

Interviews were digitally recorded and transcribed verbatim with participants ascribed a pseudonym. One researcher (NG) read and coded the transcripts to identify themes and sub themes inductively using thematic analysis with verbatim quotes selected to represent a range of views [18]. Two transcripts were reviewed by JH for verification and themes were discussed with the research team DW and AB. The Capability, Opportunity, Motivation and Behaviour (COM-B) model was used as a framework to map themes across the different groups, [19] and intervention functions with BCTs identified, based on the 40 behavioural change techniques [20]. Each theme was ascribed as a ‘barrier’ (factors that inhibit activity) or ‘facilitator’ (factors that promote activity) based on the language used by participants (Fig. 3).

## Results

The healthcare professionals included five physiotherapists, one physiotherapy technician, two occupational therapists and two nurses. Their experience in cancer ranged 2–23 years, most were located in South Wales (*n* = 8) at a variety of locations and health boards, with 2 outside Wales. The duration of the interviews ranged from 34 to 71 min.

Patient and carer interviews included four male and three female patients, age range 51–81 years. All the carers were female, age range 38–71 years. The duration of interviews ranged from 31 min to 1 h 56 min.

### Suitable physical activities

The themes identified from the interviews with HPs, patients and carers about suitable PAs are summarised in Table 1. For HPs, patients and carers homebased PA was viewed as acceptable and it was suggested that activities should be functional, individualised, flexible, and starting of low duration and intensity. The types of activities discussed included chair-based exercise, stairs, mobility and stretching. Individualisation through patient assessments, giving choice and aligning with interests, enjoyment or past activity was discussed by patients and carers. All HPs, patients and carers suggested that activities should initially be short in duration. Patients reported a range of capabilities from 5 to 15 min, and HPs said activities could be progressed, aiming for government guidelines (30 min, 5 times per week) and inactivity discouraged (Table 1).

**Table 1:** Interview Themes from healthcare professionals, patients and carers relating to suitable homebased physical activities

Themes	subthemes HPs	*Quote*	Subthemes patient/carer	*Quote*
Activities	Functional/relevant	*basic chair exercises, standing exercises standing resisted exercises and then functional stuff to do with sit to stand, walking programmes and trying to get the family involved, just trying to get some fun into the function* (Zara, physiotherapist)	Functional (mobility)	*Well I suppose I could walk up and down the stairs often.* (Ali, patient)
	Discourage inactivity	*discouraging inactivity or sedentary behaviours rather than giving specific exercises time and it may be that I would come back and do that but initially I think I would be inclined to give more functional activities.* (Sarah, physiotherapist)		*I think that walking is the most important thing is, now, starting to walk places. That's what I want to do more than anything, as I say, the rest comes later.* (Maggie, patient)
Individualised	Based on Assessment	*I would then see them in that session individually and maybe give them a bit of extra support, into sort of progressing* (Karen, physiotherapist)	Appropriate	*He is open to whatever as long as, well, so if you recommend one of those exercises and he feels that he could do it, he will* (Amelia, carer)
Flexible	Options	*it can't be a one size fits all, it needs to be tailored to individuals’ capabilities, and so whatever there needs to be, there needs to be a range of activities for various different start points.* (Heather, nurse)	Choice	*I dislike being, um, told I dislike being um, having a certain time I've got to be there or something like that I like to go my own pace get where I got to go and do what I've gotta do* (Jack, patient)
	Progress/adapt	*I try to do a bit of everything in that in that I try to do some in standing, I try to do some in sitting and even some in lying, only because I just found that some of the patients varied again like day today,* (Georgina, physiotherapist)	Interests/Past activity	*So that's how we work we know what we like and we can still do what we like that apart from biking. (Carlos, patient)*
Duration/intensity	Start small < 30mins intensity (low-mod)	*I mean I would be certainly be saying this week you know be doing 10 minutes and then each day trying to increase it gradually* (Jenny, occupational Therapist)	Duration 5−15min	*Possibly five [minutes of activity possible] (Maggie, patient). Because, be honest mum can you get from there to the shower without getting out of breath or slowing down or stopping?* (Claire, carer)

### Behaviour change theory

Interview themes were grouped by COM-B components and mapped to the *intervention functions* to promote homebased PA (Appendix 2).

Capability: An individual’s capacity to engage in the activity concerned [16]. Patients identified that physical capability was affected by symptoms including breathlessness, fatigue/tiredness, weakness, and the variability or decline in health status as well as some risks such as balance or falls:

‘whether you’d incorporate that in energy levels and that, but the consciousness of being physically weak, weaker than I was, that, you know, that's something that preys on the mind sometimes.’ (Craig, patient)

‘well it's really the breathing mainly, it's the breathing, because, it's got better yeah but I don't want to be overdoing it.’ (Jack, patient)

‘I think her balance is a bit off but that's her eyesight. I don't think it's a physical thing’ (Carol, carer)

HPs identified that nutritional status, pacing, grading activities and equipment were important for physical capability.

I say to physios that I wouldn't do too much with them, because they're not taking enough nutrition on board, so if they're, if they're not taking enough calories (Rhian, occupational therapist)

‘so they might not manage a walk down to the shop but if I say to them well let's get you standing while you clean your teeth, ok you might have a perching stool there, I want you to stand for a few minutes and then sit down because that will, you know, keep the strength in your legs so that we can then work towards you perhaps going out.’ (Jenny, occupational therapist)

Psychological capability components identified by HPs and patients as important included knowledge (what and how to do activities), attitude — including confidence — and fear of harm.

‘I mean the main thing probably I would ask for is just sort of guidance, you know, something, something that's likely to be productive and achieve an end rather than me just randomly trying to think something up or spending hours in front of the computer.’ (Craig, patient)

‘many of the lung cancer patients that came through thought they'd had this diagnosis they couldn't do anything, that they had to stop everything, thinking now we're never going to do that again, there were so many that lost their confidence so quickly.’ (Georgina, physiotherapist)

Opportunity: External factors that make possible or prompt the behaviour [16]. Physical opportunities to facilitate activity included the provision of physical resources such as examples of activities on paper or a DVD. Equipment to enable activity, e.g. a walking frame or perching stool, was thought to be useful by HPs and some patients.

I really do think having a DVD is more beneficial than having something written down because it feels like you've got company. (Carol, carer)

sometimes you have to think of a worst-case scenario, if you can’t walk you have to use equipment to get about. You know, when I use a rollator (Carlos patient)

‘if they're managing to complete the task and if they're not then we use equipment then to, to assist them with that.’ (Rhian, occupational therapist)

Social opportunities included education and advice from HPs as well as support such as prompting from carers and including carers/friends in planning and undertaking activity.

so often the intervention is based around sort of educating as well as kind of giving the exercise advice and then maybe dispelling some of the myths about what physical activity or exercise involves, (Sarah, physiotherapist)

‘I think again that's a good opportunity and, um, particularly the family members are missed out, these things are all, they're all safe and there's no reason that, that they shouldn't join in.’ (Heather, Nurse)

‘I've been bowling for years when I lived in Weston. …it's the people as well, it's very social we have a good laugh.’ (Mary, patient)

Motivation: All the brain processes that energise and direct behaviour [16]. Themes mapped to both automatic (e.g. desires, habits) and reflective (e.g. identity, beliefs, planning) motivations. HPs considered that monitoring and follow-up was beneficial, while HPs and patients suggested family/carers where important to motivate activity. This may be through prompting or participating in activities, other prompts identified by both groups were resources such as activity charts. HPs highlighted the importance of goal setting and patients preferred purposeful activities/tasks. Perceived benefits and maintaining independence were mentioned as important motives by HPs and patients. Patients additionally identified competition, enjoyment and previous PA as important for motivation.

‘The use of motivational interviewing and similar techniques to try and encourage patients to, kind of I suppose, identify what they'd like to be able to do, what their goals are, what they'd like to be able to achieve and how they think they can manage that themselves.’ (Sarah, Physiotherapist)

‘Swimming also, he went with them or with family time, so um, it's going to be a matter of, you know, but these are the most two things he used to like to do’ (Amelia, Carer)

After mapping the themes, barriers and facilitators were identified. Barriers included symptoms, suboptimal nutrition, health variability, lack of knowledge, attitude, fear, confidence and environment. Facilitators were the environment, equipment, guidance, resources, planning, social support, attitude, beliefs, goal setting, personal role, enjoyment, reinforcement and confidence.

### Intervention functions

**Fig. 4:**
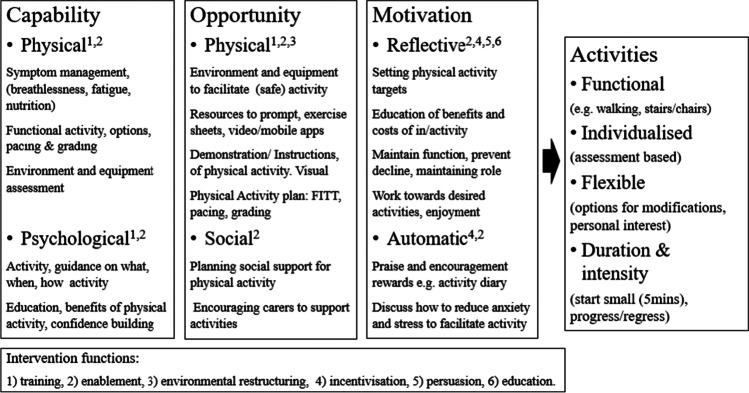
Model of principles to promote physical activity in people with lung cancer and cachexia

Based on the themes identified, six *intervention functions* (overall approaches to change behaviour) were identified with examples (Fig. 4)*.* They were *Education,* to increase knowledge and provide individualised *Training,* to guide what, when, how and why to be active. *Enablement,* to overcome barriers such as symptoms, suboptimal nutrition and the variability of disease and create a suitable environment to minimise risk and facilitate activity, as well as to build confidence and a positive attitude. This may be supported by HPs to advise and plan PA as well as prompting and participation from carers/friends to plan and undertake activity. *Environmental restructuring,* to facilitate activity modification of the home and the provision of physical resources, was suggested. *Incentivisation* through goal setting to promote and reinforce behaviour as well as *Persuasion* to emphasise perceived benefits, facilitate confidence and a positive attitude as well as *Education* to minimise fears, would motivate activity. Seventeen BCTs (more specific activities designed to change behaviour by targeting capability, opportunity and/or motivation) [13] were identified from the 40 identified by Michie et al. [20] (Appendix 3).

## Discussion

This is the first study to develop principles to promote homebased PA in patients with lung cancer and cachexia by combining views of HPs, patients and their carers, underpinned by theories of behaviour change. The focus was on physical activity, (any muscle movement that requires energy) to encompass daily activities rather than exercise (planned, structured, repetitive activity) [4]. Based on the data, specific PAs were identified including functional activities of low intensity and duration. I*ntervention functions* to promote homebased PA in lung cancer and cachexia were *Education, Training, Enablement, Environmental restructuring, Incentivisation and Persuasion*. These represent positive actions with 17 specific strategies and examples (BCTs, Appendix 3) identified to promote and facilitate PA, which could be adapted by clinical teams for local implementation.

Both HPs, patients and carers were open to the idea of homebased PA despite there being no current guidance in patients with lung cancer and cachexia. It was suggested that PA should be individualised, functional (including mobility), adaptable to individual and variable capability; starting small (low intensity and duration, 5 ~ 10 min) and potentially increased gradually. There were some concerns about risk of falls, and the need for a safe environment. The results of the HP interviews here align with the results of a study investigating the perspectives of 21 HPs on the type of lifestyle advice for cancer survivors. This interview study concluded that the optimal delivery of lifestyle advice should be (1) tailored to the individual and delivered throughout the cancer journey, (2) focused on small and achievable changes framed as part of their treatment regimen and (3) cost-effective for wide-scale implementation [21].

The results also align with our previous work [10] and the results of a survey of exercise preferences in patients with inoperable metastatic lung cancer who were motivated to exercise if given the appropriate support. Participants demonstrated low self-efficacy and preferred light intensity exercise [22]. The American Cancer Society consensus statement guidance suggests 10 min of light exercise daily is acceptable for cancer survivors experiencing severe fatigue [23]. Light activity, includes slow walking, light housework and light repetitions [24], which aligns with the views of our sample and suggestions for types and duration of PA. Indeed there is evidence that low intensity activity such as daily walking and step-counting may provide safe PA [26]. Other studies in patients with stage 4 (lung and colorectal) cancer using light intensity homebased exercise including walking and strength training [6] or a walking and Wii fit balance regimen showed potential to improve mobility, fatigue, functional status, QoL, and sleep quality [27]. It is recognised that activity intensity is dependent on individual capacity and may be guided by use of the Rate of Perceived Exertion (RPE) which has been validated in healthy people and cardiac conditions [25]. The RPE ranges from 6 to 20 with < 11 indicating light activity which equates to being able to talk during the activity [28].

### Application of the COM-B components

#### Capability

A targeted PA intervention should be designed to overcome some of the potential barriers to physical capability including suboptimal nutrition, symptoms, variability and psychological capability such as knowledge and confidence/attitude. These results align with a previous qualitative study exploring the views, attitudes and beliefs of patients and HPs towards exercise for people with lung cancer post-surgery [29]. Themes and subthemes identified were attitudes and beliefs (subtheme confidence, fear and expectancies) external factors (barriers and support), and intervention design (components, timing and individualisation) [29]. An embedded qualitative study within a pilot RCT of homebased aerobic and resistance exercise has also identified barriers including treatment side-effects and comorbidities [30]. For practical application, our findings suggest that symptom management, functional activities, consideration of the environment and equipment, as well as providing guidance and education, will promote PA capability.

#### Opportunities

Opportunities to facilitate PA in this study included the environment and equipment, a choice of resources including guidance and education of when, how and why to do activity along with a PA plan. Resources should be individualised as some patients preferred paper means while others favoured technology. A number of exercise resources, including booklets, videos and PA diaries, are available for patients with cancer, e.g. in the UK [31] and Australia [32]. However, none of these are specific to patients with cachexia. There is guidance for managing breathlessness and fatigue [33], which could be included as part of a PA intervention.

Social opportunities include family, HPs and other support networks, recognised by patients. Support groups, face-to-face and telephone follow-ups were also suggested. This aligns with results from a survey of exercise coordinators from Canada which suggested that programme enablers were patient participation (personalised care, supportive network, personal control, awareness of benefits), partnerships, advocacy and support, and appropriate program characteristics [34]. Based on this research, we encourage the use of personalised physical activity plans to include consideration of the environment, equipment, resources, demonstration of activity and social support.

#### Motivation

Themes identified within motivation included reinforcement, goals setting, perceived benefit, fear and attitude. Patients talked about enjoyment and past activities, HPs also identified maintaining role as a potential facilitator for PA interests, enjoyment or past activity. This mirrors the observations by Granger et al. [35] in patients with lung cancer, who identified the influence of enjoyment, the perceived benefits of PA, past lifestyle and return to activities of daily living in people with lung cancer. Also identified was the impact of symptoms, capacity and motivation; family and peer support; access to services and healthcare professionals. Patients suggested several factors that could improve their healthcare experience such as access to exercise professionals, information about PA in different formats; supervision from healthcare professionals and peer support [35]. We recommend setting realistic PA targets, advising on the benefits, maintaining function and enjoyable activities as well as praise and anxiety management to motivate PA.

In keeping with our study results, a previous focus group study of barriers and facilitators to exercise in 26 patients with cancer-related fatigue with a range of cancer types and severities found that exercise motivators were related to perceived exercise benefits. Barriers were related to personal factors such as cancer related symptoms or treatment, lack of motivation and environmental factors such as cost, and the lack of exercise facilities tailored to patients with cancer. Facilitators of exercise were group programmes being, supervised, individually tailored, and gradually progressed [36]. As well as the similarities there were differences, the present study finding that enjoyment was a key motivator and barriers related to lack of knowledge, attitude and fear. A systematic review of factors affecting PA in lung cancer identified patient-level factors, symptoms, comorbidities, sedentary lifestyle, mood and fear, and environmental factors. These factors should be considered to identify and develop suitable interventions [37].

Promoting a PA intervention informed by the COM-B has potential to counteract previously identified patient barriers including, symptoms, lack of knowledge the environment, lack of social support and low motivation. Therefore, personalised activity and lifestyle planning is required by cancer care workers to counter act these barriers.

### Strengths and limitations

The main strength of this study is the inclusion of healthcare professionals, patients and carers to develop principles to promote homebased physical activities based on a recognised tool the Behaviour Change Wheel. Although the sample size was small, which may limit the applicability of the findings, the age and gender of the patients who participated were representative of the UK lung cancer statistics [38]. Given the small sample size, it was not possible to confirm data saturation; however, repeating codes and themes were identified. The lead researcher is a physiotherapist with professional insight which may have influenced patients’ responses and may have provided a more positive view of physical activity in the interpretation. Although participant validation was not undertaken to minimise the burden on participants, interview scripts were reviewed by another member of the research team for quality assurance. Participants in this study were purposively selected and chose to participate, and thus, their views may differ from other healthcare professionals, patients and carers. Nevertheless, the principles may be applicable to other conditions with further development.

## Conclusion

Patients with lung cancer have low levels of PA which affects function and quality of life. Healthcare professionals are key to promoting PA; however, specific guidance to promote PA in patients with lung cancer and cachexia has not been established. Based on the insights of healthcare professionals, patients and carers, principles to promote homebased PA have been presented including six intervention functions based on behaviour change theory. These principles may assist the promotion of PA in people with lung cancer and cachexia and further research needs to evaluate the effects on physical function and emotional wellbeing.

## Supplementary Information

Below is the link to the electronic supplementary material.

Supplementary file1 (DOCX 51 KB)

## Data Availability

The datasets generated during and/or analysed during the current study are available from the corresponding author on reasonable request.
