# Supplementary material for: The promotion of homebased physical activity for people with lung cancer and cachexia, a qualitative study of healthcare professionals, patients and carers
Source: J Cancer Surviv. 2023 Apr 24;17(3):677–85. doi: 10.1007/s11764-023-01376-3 (PMC10209237; doi:10.1007/s11764-023-01376-3)
Supplement: Supplementary file 1 — Supplementary file1 (DOCX 51 KB) [file 11764_2023_1376_MOESM1_ESM.docx]

**Supplementary Material**

**The Promotion of Homebased Physical Activity for People with Lung Cancer and Cachexia, a qualitative study of Healthcare Professionals, Patients and Carers**

Journal of Cancer Survivorship

Nichola Gale (PhD, BSc) School of Healthcare Sciences, Cardiff University, Cardiff, CF14 4XN, UK

E-mail: galens@cardiff.ac.uk

**Appendix 1**

**Health Professional Interview schedule**

1. Can you tell me about your experience of supporting patients with lung cancer and weight loss to do physical activity/exercise in the home
2. How do you approach supporting people to undertake physical activity at home (what is this based on, clinical experience, local guidelines, evidence etc)
3. What kinds of Home-based physical activities are appropriate for patients with cancer cachexia. *Consider practicality, acceptability and perceived benefits* (Grande et al., 2013).

(Do you talk about ‘activities’ or ‘exercise’. What do you mean by exercise?)

1. Exercise prescription can be guided by (FITT) frequency, intensity, type, time

Is this appropriate in patients with cancer cachexia? And if so

- 1. What types of physical activities (endurance, strength, flexibility, relaxation)
  2. What intensity (degree of effort) (light, moderate, vigorous, high)
  3. How long and how often?

1. In the context of cachexia what do you consider are the benefit/risks of patients undertaking Home-based physical activity?
2. What do you find are the barriers for patients with cancer cachexia undertaking physical activity /exercise?
3. Are there any considerations for undertaking home-based physical activities (Precautions, Location, Companions, social support)
4. How can we deliver information to encourage home-based physical activity in cancer cachexia? E.g. workbook, leaflet, poster, blog, website, mobile phone application

Outcome measures

1. When treating/working with patients do you assess for cachexia? If so how? formally or informally?
2. What outcome measures do you use or do you think should/could be used to assess the effects of a home-based physical activity intervention for patients with lung cancer and weight loss?
3. What kind of outcome measures are relevant to: patients (function, Qol), health professionals (Nutrition, exercise capacity), service delivery (hospital admissions/appointments)
4. How can we assess patient’s motivation to undertake physical activity?

**Patient interview schedule**

1. Tell me about a physical activity you have done today?
2. Can you give me some examples of other kinds of physical activities that are possible for you to do, at home?
3. How important is being physical active to you? To your health?
4. For how long and how often can you be physically active each week
5. What affects the amount/types of physical activity that you do?
6. What gets in the way/limits you from being physically active or more physically active?
7. What might encourage/support you to do more physical activity at home?
8. Example of ways of increasing physical activity are walking, housework, gardening chair based activities, using stretch bands or weights.
9. What physical activities would you be willing/able to do?
10. What kind of activities would fit into your daily life
11. How would you like information about physical activity to be provided to you?

(prompt e.g. leaflet, mobile phone, video, computer etc)

- 1. When and who should give you information?

1. How can we measure the effect of doing physical activity on:
2. your health?
3. the activities you do at home?

Appendix 2

*Interview themes by COM-B components and mapped of the intervention functions to promote homebased PA*

| **COM-B and *TDF* mapping** | **Theme**  **^b^ barrier,**  **^f^ facilitator** | ***Healthcare Professionals*** | ***Patient/carer*** | **Intervention function**  and example | **Behaviour Change Technique** |
| --- | --- | --- | --- | --- | --- |
| Capability- physical | Nutrition/  Symptoms **^b^**  Variability **^b^**  Environment/  equipment **^b f^** | *I say to physios that I wouldn't do too much with them, because they're not taking enough nutrition on board, so if they're if they're not taking enough calories* (Rhian, occupational therapist)  *‘I can't imagine the patient overdoing it because, because of their, you know, what other symptoms they've got, they wouldn't have the energy to do that,* (Rhian, occupational therapist)  *Um, so they've got the flexibility of doing it as and when they feel, breaking it up through the day if that helps them. They didn't have to do all the exercises together if they could only manage to one or two having a break and then doing another couple or later in the day.* (David, Physio technician)  *so they might not manage a walk down to the shop but if I say to them well let's get you standing while you clean your teeth, ok you might have a perching stool there, I want you to stand for a few minutes and then sit down because that will, you know, keep the strength in your legs so that we can then work towards you perhaps going out.* (Jenny, occupational therapist) | *I suppose, whether you’d incorporate that in energy levels and that, but the consciousness of being physically weak, weaker than I was that, you know, that's something that preys on the mind sometimes* (Craig, patient)  *well it's really the breathing mainly it's the breathing, because, it's got better yeah but I don't want to be overdoing it.* (Jack, patient)  *So it got better and now, it's got a bit worse again?* (Amelia, carer)  *A little but I'm ok* (Max, patient)  *I think her balance is a bit off but that's her eyesight. I don't think it's a physical thing’* (Carol, Carer)  *Stairs I gasp going upstairs, I can't do them very good if, you know, if I did start off for a bath, then I got to stop halfway on the stairs to get my breath back*  (Maggie, patient) | **Enablement**  Symptom management  breathlessness management handheld fans positioning and breathing exercises, medication  **Training**  PA options, grading activities, pacing  starting small, 1-2 repetitions, lying, sitting standing n exercise  **Enablement**  Assessment of environment making safe & opportunities and equipment for activity  Perching stool, walking aids | Identifying Barriers/problem resolution  Setting graded tasks  and graded modified exercise  Environmental restructuring |
| Capability- pscychological | Knowledge **^bf^** | *I think knowledge is a big one, um, I really do I think people, people make the assumption that when you talk about exercise you talking about gyms and classes and equipment and not realising that's a simple walk at a good level is all the exercise that you could need for that day, a little incline, a step.* (David, physio technician) | *I mean the main thing probably I would ask for is just sort of guidance, you know, something, something that's likely to be productive and achieve an end rather than me just randomly trying think something up or spending hours in front of the computers.* (Craig, patient) | **Education & Training,**  Guidance/ education what when how to perform activity | Informing when and where and  How to perform the behaviour |
| Capability—*cognitive and interpersonal skills, memory, attention and decision processes, behaviour regulation* | Attitude /  Confidence **^bf^** | *many of the lung cancer patients that came through thought they'd had this diagnosis they couldn't do anything that they had to stop everything, thinking now we're never going to do that again. there were so many that lost their confidence so quickly* (Georgina, physiotherapist) | *Because walking is the natural exercise and once you get your confidence you can do it* (Magda, carer)  *It's all about confidence really* (Carlos, patient) | **Enablement**  Guidance/ education/  Reassurance of benefits of PA | Information provision (to the individual).  Others behaviour |
| Opportunity  Physical —*environmental context and resources* | Provision of equipment **^f^**  Choice of  resources **^f^** | *I think a lot of people themselves will think they haven't got the space to do it, and it's not suitable to actually do it at home and it's something you have to do in a gym environment* (Jenny, occupational therapist)  *‘its based around what they're managing to do and what they are managing at home. Not sort of assessed as in measurements, it's assessed on function and if they're managing to complete the task and if they're not then we use equipment then to, to assist them with that* (Rhian, occupational therapist)  *physio wise was chair exercises and that was a sheet of them, a sheet of standing exercises with the idea that targeting little and often so it would very much when the adverts came on when there was a change of programme to do the chair exercises* (Zara, physiotherapist) | *It's like me going up stairs. I've got some things upstairs like the Wii set up. I could have it here, but if it’s upstairs I've got to go upstairs* (Carlos, patient)  *sometimes you have to think of a worst-case scenario if you can’t walk you have to use equipment to get about. you know when I use a rollator, I think I look as though I’m 90 and I'm not. I think I look like a little old lady pushing this rollator around bit its only way I it's the only way I can get the about.* (Carlos, patient)  *Possibly put it on paper, a DVD* (Jack, patient)  *A phone because it's with me all the time, paper could get lost* (Max, patient)  *I really do think having a DVD is more beneficial than having something written down because it feels like you've got company* (Carol, carer) | **Environmental restructuring**  Ensuring a safe environment to facilitate activity  **Enablement**  Use of resources to prompt | Environmental restructuring  Training to use prompts |
|  | Guidance/ education **^f^** | *so often the intervention is based around sort of educating as well as kind of giving the exercise advice and then maybe dispelling some of the myths about what physical activity or exercise involves,* (Sarah, physiotherapist) | *Mum likes to be shown so she's not good with reading stuff down she'd rather see it being done.* (Carol, carer) | **Training** | Instruction on how to perform the behaviour  Demonstrate behaviour |
|  | Physical activity plan **^f^** | *I think that patients and family members would need advice how they should, how it goes into their day, how they should plan when those are going to be, so someone isn't as fatigued that day before they start, and how, how they can do the activity together to have a meaningful, you know, a meaningful outcome* (Heather, Nurse) | *I think, from my point of view, some sort of plan, if you know what I mean, an organised plan rather than just sort of thinking randomly I'll grab me weights and I'll do, I'll do 20, sort of, biceps lifts with my weights.* (Craig, patient) | **Enablement**  Planning, pacing, grading activity | Action Planning  Time management |
| Opportunity—*social influences* | Social support **^f^** | *I think again that's a good opportunity and um particularly the family members are missed out, these things are all, they're all safe and there's no reason that, that they shouldn't join in* (Heather, Nurse) | *you’d better giving it to xxx and then xxxx going to mum have you done it have you done it, lets do it, encouragement is more what's needed* (Claire, carer) | **Enablement** | Plan social support |
|  | Social networks **^f^** |  | *I've been bowling for years when I lived in Weston. …it's the people as well, it's very social we have a good laugh.* (Mary, patient) | **Enablement**  Negotiate patient centred goals | Plan social support |
| Motivation— Automatic *emotion* | Reinforcement **^f^** | *I think praise is important isn’t it, so they are told they’re doing well and they can see a benefit, you know to themselves, quite quickly and that in itself is self-motivating.* (Chloe, Nurse)  *I suppose if they’ve followed things like an activity diary, have they actually, you know, kind of completed it. I suppose that’s an indicator of somebody’s motivation.* (Jenny, occupational therapist) | *A star sheet a reward chart, We’ll do you a reward chart* (Sharon, carer) | **Incentivisation**  Activity diary  Provision of resources | Successful behaviour contingent rewards |
|  | Attitude **^bf^** | *I understand lung cancer can be this very nihilistic, sort of, nothing I can do, it’s the end I'm just going to fade away*  (Zara, physiotherapist) | *you can't do one thing do something else that you can or is an alternative and that's what we did with the car.* (Carlos, patient) | **Enablement**  Discussion of benefits/concerns/problem solving | Prompt self-talk |
| Motivation— Reflective *beliefs about capabilities and beliefs about consequences* | goals **^f^** | *the use of motivational interviewing and similar techniques to try and encourage patients to kind of I suppose identify what they'd like to be able to do what their goals are what they'd like to be able to achieve and how they think they can manage that themselves* (Sarah, physiotherapist) | *I do like to do something with a purpose I walk on the weekend … when I get into doing something, I like to finish it, that's my main objective.* (Jack, patient) | **Incentivisation**  Joint Goal setting | Goal setting (behaviour) |
|  | Beliefs -perceived benefit **^bf^** | *they would more motivated to engage in the program because they would knowing that was going to be treatment and they needed to be fit enough before that treatment* (Rhian, physiotherapist) | *to simply keep living and to maintain and giving indirect order to the body that this, just to sit down and just stand up. No it’s possible. resistance to the whole thing that we are going through but we still there so we should keep on doing.* (Max, patient)  *If I can, I will, it’s for my health* (Max, patient) | **Education & Persuasion**  Motivational interviewing | 1. Information provision (to the individual). |
|  | fear **^b^** | *I think one of the biggest barriers is fear um, so that might be their own fear of causing harm, causing damage especially if they're breathless they may feel that actually you know that they're not going to be able to breathe, um, or the next breath might be their last. There's quite a real strong element of fear and also it might be around their family* (Sarah, physiotherapist) | *I think it's a fear of being breathless because it doesn't take very long for, for her to start being tight chested and then it goes into like anxiety and she'll just stop because I think she stops herself from doing a lot of things because she doesn't want to go too far in case she can't get back and it's just the fear of being stranded* (Sharon, carer) | **Education/**  Guidance/ education Pacing grading | Stress management |
|  | Intentions /role  **^f^** | *in patients with lung cancer they are normally rapidly deteriorating, so they're trying to graft some sort of role in their life, and I don't want to, sort of, loose everything* (Rhian, occupational therapist) |  | **Incentivisation**  Focus on Maintaining roles | Action planning |
|  | Enjoyment past activity **^f^** |  | *Just sitting on the swings or pushing them on the swings I think that would motivate her as well because used to enjoy that in the summer didn't you just sitting there.* (Sharon, carer) | **Incentivisation**  Work towards desired activities | Focus on past success |

^b^ = barrier ^f^ = facilitator

Insertions to clarify topic content are denoted by square brackets. The removal of irrelevant information within the quotes is denoted by "....".

Appendix 3 Behaviour Change Techniques and examples to promote homebased PA in Lung cancer and cachexia

| Identifying Barriers/problem resolution   - Symptom management, Breathlessness: Positioning, breathing exercises, handheld fans, medication - Nutrition: Encourage frequent eating   Information provision (to the individual).   - Guidance/ education/ of benefits of PA cost of inactivity   Goal setting (behaviour)   - Setting target behaviour short erm and longer term e.g. daily mobility, aim to walk to bus stop   Action Planning   - Create Personalised Physical Activity plan: modified activities, pacing and grading (FITT) (see activities)   Setting graded tasks   - Start with easy tasks and increase difficulty/adapt as able (e.g. 2 >5 sit to stands, 2- 5mins)   Informing when, and where to perform behaviour   - Education/guidance on when and where to perform activity (see activities) (consider prompts in the home, symptoms, fatigue)   Instruction on how to perform the behaviour   - Verbal/ written PA Instructions   Demonstrate behaviour   - Visual examples of PA’s, in person, video, mobile phone application   Training to use prompts   - Provide resources to prompt, exercise sheets, video/mobile apps. Home prompts activities while adverts are on TV   Environmental restructuring   - Environment and equipment, assess for safety & opportunities for activity e.g. Rugs, space, perching stool, walking aids   Successful behaviour contingent rewards   - Praise and encouragement rewards may include activity diary - To maintain role e.g. get dressed, make food, drink   Focus on past success   - Work towards desired activities, enjoyment e.g.walk around the garden, to the shop/park   Time management   - Identify suitable time for PA, balance time/energy/life commitments - Prioritize tasks, consider individual limits, ask for help, focus on positive actions   Stress management   - Reduce anxiety and stress to facilitate PA, appropriate & flexible PA,   Plan social support   - Planning to Involve carers/friends in activity, Encouraging carers to support /prompt PA - Planning social activity for physical activity e.g. meeting with family, bowling   Others behaviour   - provide examples of similar patients achieving PA   Prompt self-talk   - Encourage positive thinking/ problem solving to facilitate activity |
| --- |
